# Protective Effect of Electroacupuncture on Chemotherapy-Induced Salivary Gland Hypofunction in a Mouse Model

**DOI:** 10.3390/ijms241411654

**Published:** 2023-07-19

**Authors:** Thanh-Hien Vu Nguyen, Kuo-Chou Chiu, Yin-Hwa Shih, Chung-Ji Liu, Tran Van Bao Quach, Shih-Min Hsia, Yi-Hung Chen, Tzong-Ming Shieh

**Affiliations:** 1 Graduate Institute of Acupuncture Science, China Medical University, Taichung 40402, Taiwan; 2 School of Dentistry, China Medical University, Taichung 40402, Taiwan; 3 Division of Oral Diagnosis and Family Dentistry, Tri-Service General Hospital, National Defense Medical Center, Taipei 11490, Taiwan; 4 Department of Healthcare Administration, Asia University, Taichung 41354, Taiwan; 5 Department of Oral and Maxillofacial Surgery, MacKay Memorial Hospital, Taipei 10449, Taiwan; cjliu3229@gmail.com; 6 School of Nutrition and Health Sciences, Taipei Medical University, Taipei 110301, Taiwan; 7 Nutrition Research Center, Taipei Medical University Hospital, Taipei 110301, Taiwan; 8 Chinese Medicine Research Center, China Medical University, Taichung 40402, Taiwan; 9 Department of Photonics and Communication Engineering, Asia University, Taichung 41354, Taiwan

**Keywords:** xerostomia, salivary hypofunction, chemotherapy, 5-fluorouracil, inflammation, acupuncture

## Abstract

Radiotherapy and chemotherapy can impair salivary gland (SG) function, which causes xerostomia and exacerbate other side effects of chemotherapy and oral infection, reducing patients’ quality of life. This animal study aimed to assess the efficacy of electroacupuncture (EA) as a means of preventing xerostomia induced by 5−fluorouracil (5−FU). A xerostomia mouse model was induced via four tail vein injections of 5−FU (80 mg/kg/dose). EA was performed at LI4 and LI11 for 7 days. The pilocarpine-stimulated salivary flow rate (SFR) and salivary glands weight (SGW) were recorded. Salivary immunoglobulin A (SIgA) and lysozyme were determined via enzyme-linked immunosorbent assay (ELISA). SG was collected for hematoxylin and eosin staining to measure acini number and acinar cell size. *Tumor necrosis factor*-*α (TNF*-*α)*, *interleukin*-*1β (IL*-*1β)*, and *aquaporin 5 (AQP5)* mRNA expressions in SG were quantified via RT-qPCR. 5−FU caused significant decreases in SFR, SGW, SIgA, lysozyme, *AQP5* expression, and acini number, while *TNF-α* and *IL-1β* expressions and acinar cell size were significantly increased. EA treatment can prevent 5−FU damage to the salivary gland, while pilocarpine treatment can only elevate SFR and AQP5 expression. These findings provide significant evidence to support the use of EA as an alternative treatment for chemotherapy-induced salivary gland hypofunction and xerostomia.

## 1. Introduction

Saliva plays a crucial role in oral health and gastrointestinal system homeostasis. Mechanical cleansing, salivary composition, and antimicrobial activity of saliva help to prevent oral infections and maintain tooth integrity. Salivary production that is insufficient in both quantity and quality, as assessed based on the salivary flow rate and composition, to maintain oral homeostasis and physiological function is known as salivary gland dysfunction or hypofunction, and it usually leads to dry mouth or xerostomia, i.e., a subjective feeling of oral dryness. There is a connection between the malfunction of the salivary glands and various diseases and conditions, such as head and neck cancer, salivary gland infection, sialadenitis or duct obstruction, Sjögren’s syndrome, and other conditions, including cystic fibrosis, uncontrolled diabetes, aging, etc. [1].

Xerostomia and oral mucositis are two of the most common adverse reactions to radiotherapy and chemotherapy. The prevalence of chemotherapy-induced xerostomia is around 50% according to Jensen et al. (2010) or 59% according to Wilberg et al. (2014), though it can be altered based on treatment regimens and underlying conditions. Some patients may suffer xerostomia during and following chemotherapy from 1–2 weeks up to 6–12 months, while others are not affected to any noticeable extent [2,3]. Although chemotherapy-induced xerostomia was less concerning than that induced via radiotherapy, it can exacerbate oral mucositis [4], and, in the case of systemic chemotherapy regimens, it can also occur concurrently with mucositis, which affects the gastrointestinal tract and oral cavity, causing significant discomfort, anorexia, and malnutrition. These complications predispose patients to oropharyngeal candidiasis, dental decay, and other gastrointestinal infections; worsen cancer pain during and after cancer treatment; and necessitate reducing chemotherapy and delaying cancer treatments. These strategies would have a considerable impact on the patient’s quality of life and overall survival rate [5]. Hence, treatment is essential, both for oral health maintenance and when provided as palliative care. It also helps improve quality of life, particularly for those patients who already have other serious underlying conditions, e.g., gastrointestinal diseases, oral infections, and other conditions that decrease salivary secretion before cancer treatment.

Salivary glands (SG) are histologically made up of acini and ductal structures. While the ducts are mostly comprised of intercalated and striated cells, the acinus is made up of several acinar cells arranged concentrically around a central lumen. The acinus is surrounded by a connective tissue matrix, which incorporates the basal lamina, though it does not insert itself between individual acinar cells [6]. Serous acini of the submandibular gland (SMG) contain strongly stained, polarized epithelial cells with a granular appearance [7]. Acini are the basic secretory unit of the SG, being responsible for the salivary production function of the salivary glands, while the ducts modify the fluid secreted by the acini [8,9].

Management strategies for xerostomia and mucositis induced via cancer therapies can include prevention or minimization of symptoms, including intensity-modified radiotherapy (IMRT), muscarinic agonist stimulation (pilocarpine or cevimeline), oral rinses, anti-inflammatory agents (benzydamine mouthwash), and oral mucosal lubricants (saliva substitutes). However, these treatments are not always effective, and some of them even cause other adverse reactions. Therefore, new treatment options were introduced, including Amifostine [10], basic fibroblast growth factors (bFGF) [11], Gliclazide [12], and low-level laser therapy and acupuncture [13,14].

Several studies were conducted to investigate the effectiveness of acupuncture in treating xerostomia. For example, reliable cancer therapies (RCT) developed by Garcia et al. (2019), whose study included 339 patients, showed that acupuncture resulted in significantly fewer and less severe xerostomia symptoms 1 year after treatment compared to standard care [15]. A systematic review conducted by Assy and Brand (2018) reported that some studies suggested that acupuncture could be an effective treatment, while others were inconclusive due to insufficient evidence. Thus, further studies are necessary to determine the potential benefits of acupuncture [16]. A previous RCT study found that acupuncture at LI2 caused an increase in saliva production, which was associated with neuronal activations [17]. The Large Intestine (LI) meridian is connected to the large intestine, lungs, lower teeth, mouth, nose, and face; according to meridian theory, LI can be a potential meridian for treating salivary ailments [18]. And, although different clinical trials of acupuncture for radiotherapy- or chemotherapy-induced xerostomia have used different acupoints, LI2 and LI4 acupoints were still frequently used, as their utilization rates were 35.29%, being second in terms of utilization to ST36 (47.06%) [19]. In October 2021, *Nature* published an original research article that demonstrated that electroacupuncture (EA) stimulation mediates the anti-inflammatory effect [20]. It has aroused great concern and extensive discussion in the field of acupuncture. Nonetheless, the efficacy of acupuncture for the protection and treatment of salivary gland hypofunction conditions, especially the pre-clinical aspects and the mechanisms underlying them, remains unclear.

This animal study aims to assess the efficacy of electroacupuncture (EA) in preventing xerostomia, salivary gland hypofunction, and inflammation, as well as the decrease in SG’s acini number and Aquaporin 5 (AQP5) induced by 5-fluorouracil (5−FU).

## 2. Results

### 2.1. Body Weight, Accumulated Food, and Water Consumption Were Decreased in 5−FU-Injected Mice

**Figure 1:**
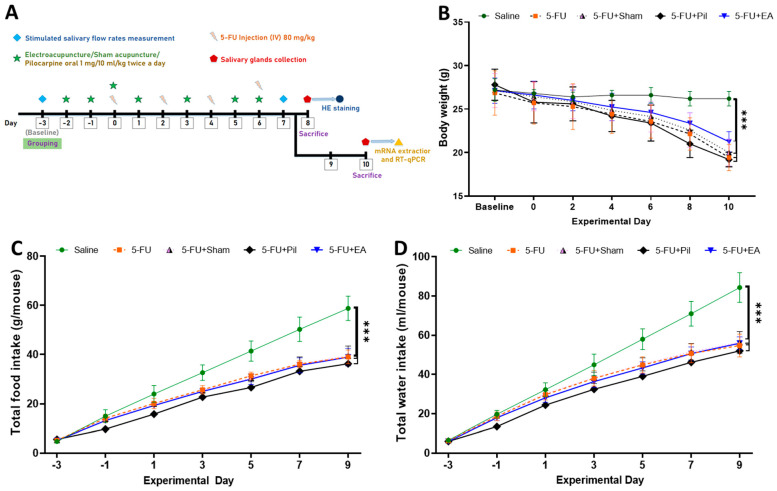
Experimental schedule and changes in body weight based on accumulated food and water consumption. (**A**) Schematic diagram of experimental timeline. (**B**) A rapid decrease in body weight of all 5−FU-injected mice occurred relative to the saline mice. 5−FU + EA mice have a slightly lower decrease in body weight among the 5−FU-injected mice, but the difference is not statistically significant. Changes in accumulated food (**C**) and water (**D**) consumption. Food and water consumption were measured from the baseline day to day 10. Data are expressed as mean ± SD; *** *p* < 0.001; data on day 10 were analyzed using One-way ANOVA with Tukey’s multiple comparisons test.

The first tail vein injection of 5−FU was regarded as day 0, and the days before and after were represented by − or +, respectively. Saliva flow rate measurements were performed on days −3 and 7. On days 0, 2, 4, and 6, 5−FU was injected into the tail vein. Electroacupuncture stimulation was performed on days −2, −1, 0, 1, 3, 5, and 6. On day 8, the mice were sacrificed for salivary gland tissue sections and H&E staining, and another batch of mice was sacrificed on day 10 to separate the salivary gland tissue and extract RNA (Figure 1A). The experiment was divided into 5 groups, namely the Saline, 5−FU, 5−FU Sham, 5−FU + Pil, and 5−FU + EA groups. For further details, see Section 4.1. 5−FU caused a rapid decrease in the body weight of all 5−FU-injected mice relative to the saline-injected mice. As Figure 1B shows, 5−FU, 5−FU + Sham, and 5−FU + EA mice experienced a significant decrease in body weight (day 10) compared to Saline mice. Although the body weight of 5−FU + EA mice decreased to a slightly lesser extent than that of the 5−FU and 5−FU + Sham mice, the difference was not statistically significant.

Correspondingly, the accumulated food and water consumption of all 5−FU-injected mice were significantly lower than those of Saline mice (Figure 1C,D). Food and water consumption were measured with respect to each group from the baseline day to the day of sacrifice.

### 2.2. EA and Pilocarpine Prevented the Stimulated Salivary Flow Rate Decrease, but Only EA Treatment Diminished the Salivary Gland Weight Decrease Caused by 5−FU

**Figure 2:**
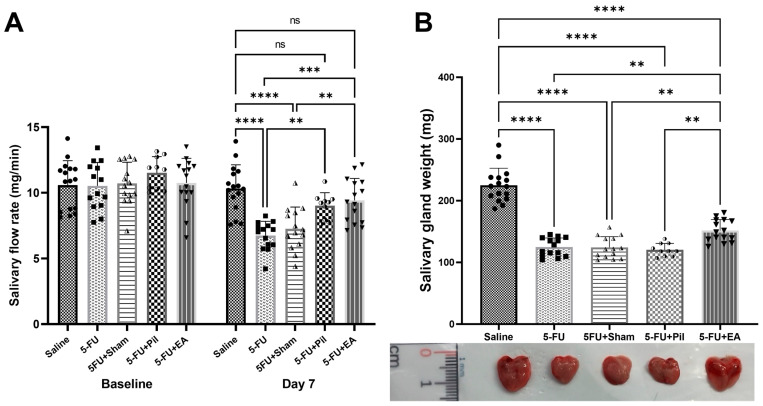
Effect of EA on 5−FU-induced salivary flow rates and salivary gland weight reduction. (**A**) Salivary flow rates (SFR) in Saline, 5−FU, 5−FU + Sham, 5−FU + Pil, and 5−FU + EA groups. Baseline (day −3), and day 7 of 5−FU induction and therapies. (**B**) Salivary gland weight (SGW) in Saline, 5−FU, 5−FU + Sham, 5−FU + Pil, and 5−FU + EA groups. Baseline, day −3, and day 7 of 5−FU induction and therapies. Below the chart, there is a photo of the salivary glands of each group. Scale ruler: 15 mm. Data are expressed as mean ± SD; ** *p* < 0.01, *** *p* < 0.001; **** *p* < 0.0001; ns, no significance; n = 10–16 in each group. One-way ANOVA with Tukey’s multiple comparisons test.

The stimulated salivary flow rate (SFR) was measured on the baseline day and day 7. There was no significant difference in SFR on the baseline day between groups. On day 7, SFR of 5−FU and 5−FU + Sham were significantly decreased compared to those quantities of the Saline and 5−FU + EA groups. Although there were slight decreases in the SFR of 5−FU + EA and 5−FU + Pil compared to that of the Saline group, the differences were not statistically significant. There was also no significant difference in the SFR between EA and pilocarpine treatment (Figure 2A).

The salivary gland weight (SGW) measured on day 10 of the experiment shows that all 5−FU-injected groups experienced a significant decrease in SGW compared to the Saline group, the SGW of which was 1.8 times bigger than those of 5−FU, 5−FU + Sham, and 5−FU + Pil groups. However, the SGW of 5−FU + EA was still significantly increased compared to 5−FU, 5−FU + Sham, and 5−FU + Pil groups (Figure 2B).

### 2.3. Observation of SMG Hematoxylin and Eosin Staining: EA Treatment Prevented the Decrease in Acini Number and the Increase in Acinar Cell Size Induced by 5−FU

**Figure 3:**
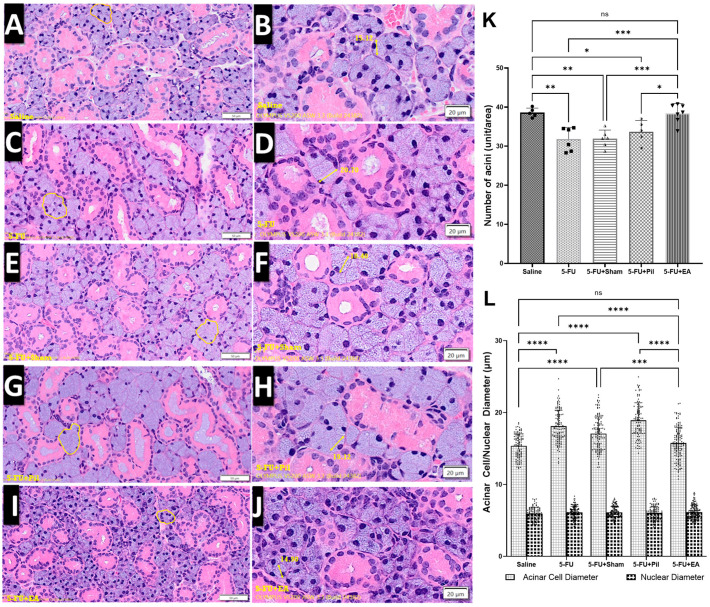
Histological changes in hematoxylin and eosin staining of submandibular glands. Images of Saline (**A**,**B**), 5−FU (**C**,**D**), 5−FU + Sham (**E**,**F**), 5−FU + Pil (**G**,**H**), and 5−FU + EA (**I**,**J**) groups show representative areas from H&E staining tissue slides of SMG. The irregular yellow marker areas (**A**,**C**,**E**,**G**,**I**; ×200 magnification, scale bar: 50 µm) and double-headed arrows (**B**,**D**,**F**,**H**,**J**; ×400 magnification, scale bar: 20 µm) indicate the secretory acini and acinar cell diameters (expressed as µm), respectively. (**K**) Differences in secretory acini numbers in the H&E staining of the submandibular gland (SMG) between experimental groups. (**L**) Comparison between acinar cell and nuclear sizes. Data are expressed as mean ± SD; * *p* < 0.05; ** *p* < 0.01; *** *p* < 0.001; **** *p* < 0.0001; ns, no significance; n = 5–7 in each group. One-way ANOVA with Tukey’s multiple comparisons test.

Figure 3A–J shows representative areas of hematoxylin and eosin (H&E) staining of SMG tissue slides from five experimental groups. The numbers of secretory acini in the 5−FU, 5−FU + Sham, and 5−FU + Pil groups were significantly fewer than those of the Saline and 5−FU + EA groups (Figure 3K). Although the number of acini in the 5−FU + Pil group was slightly increased compared to those of 5−FU and 5−FU + Sham groups, the differences were not statistically significant.

In contrast, the acinar cell sizes of the 5−FU, 5−FU + Sham, and 5−FU + Pil groups were significantly bigger than those of the Saline and 5−FU + EA groups, as shown in Figure 3L. The acinar cell sizes of 5−FU + Pil were the biggest among all groups. Meanwhile, there were no significant differences in acinar cell sizes between the Saline and 5−FU + EA groups. Moreover, no significant difference in acinar nuclear diameters was recorded between all groups.

In summary, the 5−FU, 5−FU + Sham, and 5−FU + Pil groups have fewer acini but bigger acinar cell sizes than the Saline and 5−FU + EA groups.

### 2.4. EA Diminished the Decrease in Salivary IgA Secretion Rates and Lysozyme Activity Caused by 5−FU

**Figure 4:**
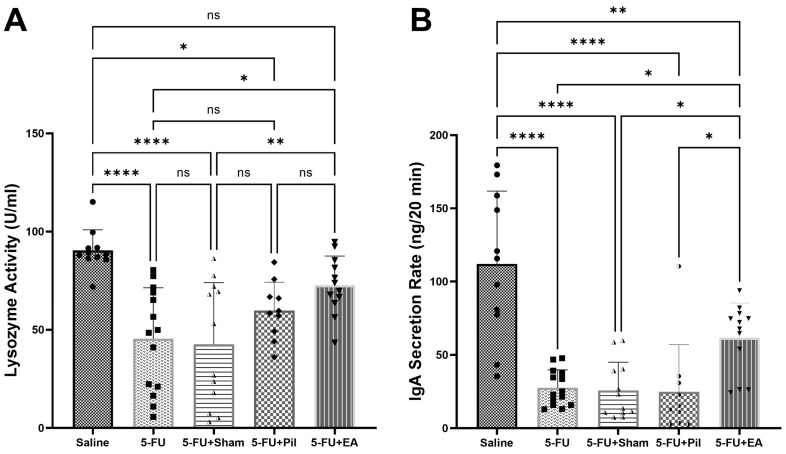
EA diminished the decrease in salivary IgA secretion rates and lysozyme activity caused by 5−FU. (**A**) Lysozyme activity of Saline, 5−FU, 5−FU + Sham, 5−FU + Pil, and 5−FU + EA groups. (**B**) Salivary IgA (SIgA) secretion rates of Saline, 5−FU, 5−FU + Sham, 5−FU + Pil, and 5−FU + EA groups. Data are expressed as mean ± SD; * *p* < 0.05; ** *p* < 0.001; **** *p* < 0.0001; ns, no significance; n = 10–14 in each group. One-way ANOVA with Tukey’s multiple comparisons test.

The salivary flow rate and salivary compositions were the indicators of the salivary glands’ function. The salivary lysozyme and SIgA were assessed to measure salivary composition [21,22]. Results obtained via the lysozyme activity assay showed a significant decrease in the lysozyme activity of the 5−FU, 5−FU + Sham, and 5−FU + Pil groups compared to the Saline group. The lysozyme activity of the 5−FU + EA group showed a significant increase compared to the 5−FU and 5−FU + Sham groups. Meanwhile, with pilocarpine treatment, the lysozyme activity of the 5−FU + Pil group was higher than those of 5−FU and 5−FU + Sham groups, but still lower than EA treatment, although the differences were not statistically significant (Figure 4A). Results obtained via the salivary IgA (SIgA) secretion rate assay showed a similar pattern, in which 5−FU caused a significant decrease in the SIgA secretion rate compared to saline injection. However, with EA treatment, the SIgA secretion rate of the 5−FU + EA group showed a significant increase compared to the 5−FU, 5−FU + Sham, and pilocarpine treatment groups (Figure 4B).

### 2.5. EA Attenuated the 5−FU-Induced Pro-Inflammatory Cytokines (Tumor Necrosis Factor-α and Interleukin-1β) Expressions in the Salivary Gland

**Figure 5:**
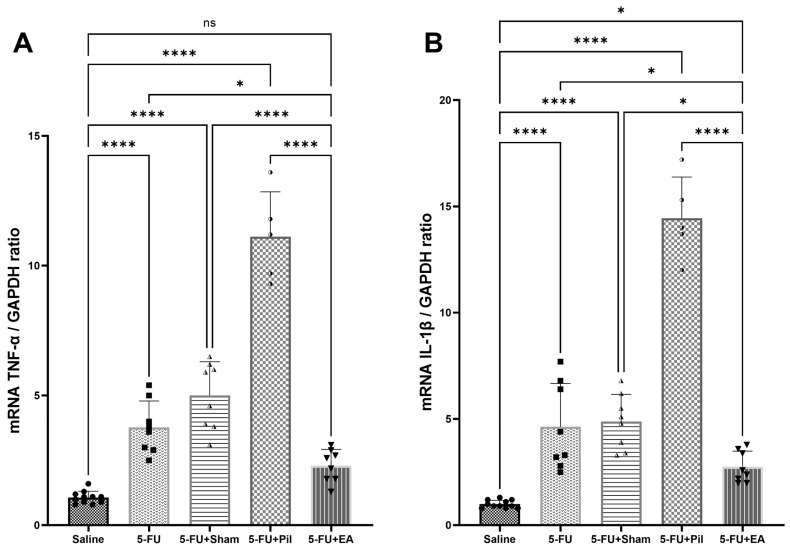
EA reversed the increase in pro-inflammatory cytokine genes induced by 5−FU in salivary gland tissue. (**A**) *TNF-α* mRNA level in SG of the Saline, 5−FU, 5−FU + Sham, 5−FU + Pil, and 5−FU + EA groups. (**B**) *IL-1β* mRNA level in SG of the Saline, 5−FU, 5−FU + Sham, 5−FU + Pil, and 5−FU + EA groups. SG tissues for RT-qPCR were collected on day 10. Data are expressed as mean ± SD; * *p* < 0.05, **** *p* < 0.0001; ns, no significance; n = 5–11 in each group. One-way ANOVA with Tukey’s multiple comparisons test.

Intraperitoneal injection of lipopolysaccharide (LPS) into mice induced the expression of *tumor necrosis factor-α* (*TNF-α)* and *interleukin-1β (IL-1β)* mRNA in the submandibular gland [23]. The chemotherapy 5−FU treatment in hamsters induced the expression of *IL-1β* and *TNF-α* levels in submandibular glands [21]. Thus, the expression of *IL-1β* and *TNF-α* mRNA were used for pro-inflammatory cytokines analysis in the SG. As shown in Figure 5A,B, *TNF-α* and *IL-1β* mRNA expression in SG tissue of the 5−FU, 5−FU + Sham, and 5−FU + Pil groups were significantly increased compared to those of the Saline and 5−FU + EA groups. Although SG’s *TNF-α* mRNA expression in the 5−FU + EA group was higher than that of Saline group, the difference was not statistically significant. In contrast, SG’s *IL-1β* mRNA expression of 5−FU + EA was still significantly increased compared to that of the Saline group (*p* < 0.05). Thus, 5−FU’s toxicity induced the inflammatory process using SG, though EA treatment helped to attenuate that effect. Unexpectedly, pilocarpine treatment in 5−FU-injected mice caused a significant increase in *TNF-α* and *IL-1β* mRNA expression in SG.

### 2.6. Pilocarpine and EA Treatment Reversed the Decrease in AQP5 mRNA Expression Induced by 5−FU in the Salivary Glands

**Figure 6:**
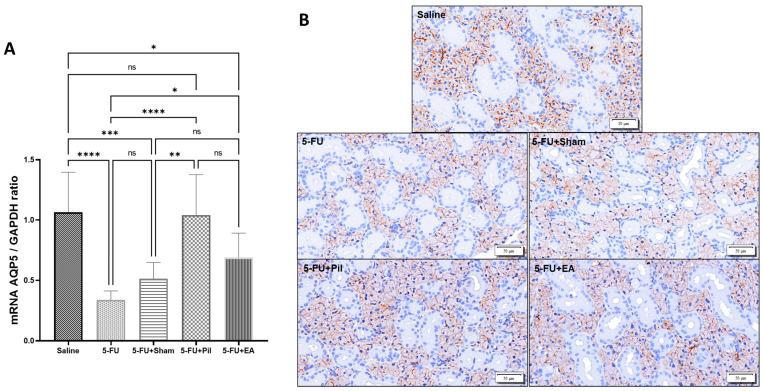
Pilocarpine and EA treatment increased *AQP5* mRNA and protein expression in the salivary gland. (**A**) *AQP5* mRNA level and (**B**) AQP5 protein expression and location in SG of Saline, 5−FU, 5−FU + Sham, 5−FU + Pil, and 5−FU + EA groups. SG tissues for RT-qPCR and *immunohistochemistry* were collected on day 10. Data are expressed as mean ± SD; * *p* < 0.05; ** *p* < 0.01; *** *p* < 0.001; **** *p* < 0.0001; ns, no significance; n = 5–11 in each group. One-way ANOVA with Tukey’s multiple comparisons test. In the tissue, brown represents the location of AQP5 protein, and the intensity of the color represents the amount of AQP5 protein.

Aquaporin-5 (AQP5) is a water channel protein that is primarily found on the apical membrane of acinar cells of serous acini. It regulates water permeability in acinar cells, affecting the flow rate and ionic composition of saliva. One study reported that AQP5-deficient mice tended to produce less saliva with a hypertonic and viscous consistency than wild-type mice [24,25]. In this current study, *AQP5* mRNA expression in SG of the 5−FU, 5−FU + Sham, and 5−FU + EA groups were significantly decreased compared to that of the Saline group, indicating that 5−FU caused a significant reduction in *AQP5* expression in SG. However, EA treatment reduced that effect through a significant increase in *AQP5* mRNA expression compared to the 5−FU group. On the other hand, pilocarpine treatment significantly increased *AQP5* mRNA expression in the 5−FU + Pil group compared to those in the 5−FU and 5−FU + Sham groups. Although *AQP5* mRNA expression in the 5−FU + Pil group was higher than that of the 5−FU + EA group, the difference was not statistically significant (Figure 6A). AQP5 protein expression and location were performed using an immunohistochemistry (IHC) assay. The patent of AQP5 protein expression was consistent with AQP5 mRNA expression in each group (Figure 6B).

## 3. Discussion

In this study, a mouse model of chemotherapy-induced salivary gland hypofunction was successfully established via 5−FU tail vein injection. 5−FU is a fluoropyrimidine commonly used as an antineoplastic agent to treat multiple solid tumors, including colon, rectal, breast, gastric, pancreatic, ovarian, bladder, liver, and head and neck cancers [26]. In humans, 5−FU is capable of reducing the saliva flow rate which results in xerostomia, modification of salivary pH, ionic components, and salivary enzymes [27]. In this study, the tail vein injection of 5−FU caused significant decreases in SFR and SGW, as well as salivary compositions (lysozyme and IgA). Food and water consumption, as well as body weight, rapidly decreased in parallel. Histopathological observations showed a significant reduction in the number of acini and hypertrophic acinar cells on SMG. In agreement with our data, a study of morphological changes in the salivary gland and colon of BALB/c mice caused by tail vein injection of 5−FU reported a decrease in SFR and SGW [28]. In addition, Bertolini et al. (2017) found that tail vein injection of 5−FU alone also inflicted histopathological and inflammatory responses on the murine oral mucosa, while intraperitoneal injection without noxious stimuli did not inflict such responses [29]. 

This current animal study makes a significant contribution to the research field of the protective effect of EA against chemotherapy toxicity, specifically focusing on salivary gland hypofunction and xerostomia. Several studies were conducted to evaluate the effect of acupuncture on xerostomia and salivary gland hypofunction. Nearly all of these studies were clinical trials in which manual, auricular, or electro-acupuncture had been used to treat xerostomia following cancer therapy. These studies found that acupuncture may be an effective treatment that improves the patients’ xerostomia conditions and salivary pH, though any advantage of true acupuncture over sham acupuncture was not apparent [30,31]. In addition, there were studies of healthy subjects that pointed out that acupuncture increased the production of saliva and salivary IgA [17,32]. Meanwhile, our study provides compelling evidence to support the protective effect of EA against chemotherapy-induced salivary gland hypofunction in an animal model, shedding light on unexplored aspects, including the capacity of EA to restore salivary lysozyme activity. Notably, some studies reported that transcutaneous electrical stimulation, when given simultaneously with radiotherapy, was effective in treating irradiation-induced xerostomia [33]. In contrast, the sham acupuncture (performed via electrical stimulating lateral deltoid muscles) performed in this study only had a limited or null effect, highlighting the important role of needle placements and acupoints. 

EA treatment’s protection against the decrease in the acini numbers of SGW and SMG induced by 5−FU is a newly discovered effect, and fresh evidence supports the potential benefits of EA. The decrease in quantity and quality of saliva production is a result of the decrease in SGW and the number of secretory acini caused by 5−FU. However, EA performed at LI4 and LI11 in tandem with 5−FU administration can reverse those reductions, which results in the effect of 5−FU on salivary production being diminished. In contrast, sham acupuncture and pilocarpine treatment did not have those effects, and their salivary glands remained smaller in weight and fewer in acini number but had larger acinar cell sizes than 5−FU + EA. These histological findings are in line with those of Fujiwara et al. (2022) regarding the 5−FU effect on SG [34]. Thus, the results from our study indicate that EA at LI4 and LI11 can aid in the recovery of 5−FU-damaged salivary glands. Acinar cell hypertrophy’s cause, however, remains unexplained. Our theory to explain this phenomenon is that the salivary glands’ effort to compensate for their function being impaired by 5−FU toxicity is its root cause. Interestingly, the sizes of acinar cells in the 5−FU + Pil group were the largest among the experimental groups. In contrast, a study reported that isoproterenol can increase acinar cell sizes, while pilocarpine administration alone in healthy rats cannot increase acinar cell sizes [35]. Studies also reported that simultaneous administration of pilocarpine during radiation or midazolam treatments could enhance the proliferation of SG acinar cells in rats [36,37]. Hence, we suggest the hypertrophy of acinar cells under 5−FU’s effect was enhanced in combination with pilocarpine treatments. Moreover, the hypertrophic acinar cell plus M3 muscarinic agonist activation mechanism is one function that helps to maintain the SFR and increase the AQP5 expression in pilocarpine-treated mice.

This study shows that EA and pilocarpine treatments prevented the reduction in SG‘s AQP5 expression induced by 5−FU. The reduction in salivary flow rates under pilocarpine stimulation in AQP5-null mice compared to normal mice indicates that AQP5 plays an important role in stimulated salivary secretion, though its role in basal salivary secretion remains unclear [38]. Moreover, AQP5 expression is downregulated in response to radiation therapy, which can lead to a decrease in salivary flow rates and the development of xerostomia. In line with our data, one study found that pilocarpine improved SMG’s AQP5 expression in irradiated mice [39], while another study found AQP5 was decreased in SG of pilocarpine-administered healthy rats [35]. However, there was no previous research that investigated the effect of acupuncture on AQP5 expression in the SG. According to this study, the protective effect of EA on the number of SMG acini may be associated with the increase in AQP5 compared to 5−FU, though AQP5 is not the main reason for the existence of the mechanism behind the increased salivary production of EA treatment.

We observed 5−FU caused a significant increase in the expression of pro-inflammatory cytokines TNF-α and IL-1β gene in the SG. Previous research has demonstrated that 5−FU could elevate inflammatory mediators. According to Bomfin et al. (2017), 5−FU boosted the salivary glands’ inflammatory response, permitting the release of pro-inflammatory cytokines and an influx of more inflammatory cells [21]. Evidence has been found that suggests that the inflammatory process plays an important role in conditions such as Sjögren syndrome, salivary gland infections, obstructive disease, and radiotherapy- and chemotherapy-induced xerostomia [40,41]. Although there may not be significant tissue damage in the early stage of the disease, it has been proposed that the inflammatory cytokines released can affect acinar cell secretory function. Loss of secretory function has been attributed to infiltration by inflammatory cells [42]. Many studies have clarified the anti-inflammatory effect of acupuncture on multiple tissues and organs. Acupuncture at ST25, BL60, SP6, GV20, PC6, LI4, LI11, and other points, such as ST36, was found to have an anti-inflammatory effect, which reduced TNF-α and IL-1β levels in serum, brain, spinal cord, lung, cardiac muscles, ankle joint, and hind paw tissues [43,44,45,46,47]. In this study, we found that EA at LI4 and LI11 also had an anti-inflammatory action on SG, since it reduced the increase in TNF-α and IL-1β expression induced by 5−FU. Pilocarpine administration at a dosage of 1 mg/kg twice a day, surprisingly, was found to significantly increase the expression of TNF-α and IL-1β. This result suggests that 1 mg/kg is not the optimal dose for pilocarpine treatment in a mouse model of chemotherapy-induced xerostomia.

Considering these results, it is clear that EA can provide a non-pharmacological therapy alternative for xerostomia and salivary gland hypofunction conditions, which often require long-course therapy. Long-course pharmaceutical treatment for these conditions is likely to cause significant adverse effects. In past studies, excessive sweating, stomach upset, and dizziness were most commonly reported during pilocarpine treatment [48]. Nausea, vomiting, and hypotension were common adverse effects of amifostine treatment, particularly when provided via intravenous administration, which sometimes led to treatment discontinuation, particularly in chemotherapy-receiving patients [49,50]. In contrast, EA is an affordable, minimally invasive, and eco-friendly treatment. Adverse events related to acupuncture treatment can be none or mild, including temporary pain, minor bruising, or bleeding at the sites of acupoints [19,51]. The results of this study provide clear evidence to support the beneficial effects of EA on chemotherapy-induced xerostomia, though the pathway behind the protective effect of EA against 5−FU toxicity on the salivary glands remains unclear, requiring further elucidation.

## 4. Materials and Methods

### 4.1. Experimental Animals

12- to 13-week-old male C57BL/6 mice (National Laboratory Animal Center, NARLabs, Taipei, Taiwan), which weighed 22–29 g, were used in this experiment. Mice were housed under 12-h light–dark cycles with free access to food and water. All procedures conducted obtained approval from the Institutional Animal Care and Use Committee of China Medical University (protocol number: CMUIACUC-2022-159).

### 4.2. Study Design and Experimental Groups

The animals were randomly divided into 5 groups:

(1) 5−FU group: 5−FU injection, and anesthesia was only given on treatment days.
(2) Sham acupuncture group: 5−FU injection with sham acupuncture treatment (5−FU + Sham).
(3) Electroacupuncture (EA) group: 5−FU injection with EA treatment (5−FU + EA).
(4) Pilocarpine—positive control group: 5−FU injection and 1 mg/10 mL/kg pilocarpine (Sigma-Aldrich, St. Louis, MO, USA) given orally twice per day on the same days as EA sessions (5−FU + Pil); the dose was chosen based on a published study [52], and other groups were instead given distilled water. The use of pilocarpine as an effective prophylactic and treatment medication for xerostomia was reported in the previous study [53].
(5) Control group: administered PBS only, and anesthesia was only given on treatment days (Saline)

There were 10–16 animals assigned to each group. At baseline, each group had nearly equal means for body weight and salivary flow rate. The experimental schedule of this study is shown in Figure 1A.

### 4.3. Mouse Model of 5−FU-Induced Xerostomia

Mice from all 5−FU-injected groups were given four intravenous administrations of 5−FU (Sigma-Aldrich, St. Louis, MO, USA; 80 mg/kg) on days 0, 2, 4, and 6; the protocol used was modified on the basis of a previously published study [21]. Body weight, food, and water consumption were measured once every 48 h from baseline to day 10. The mice were sacrificed on either day 8 or 10, their salivary glands (submandibular gland and sublingual gland) were collected, and the wet weight of SG was measured. Next, the glands were divided into two sides: one side was stored at −80 °C for later biochemical analysis, and the other side was fixed in 10% neutral buffered formalin for histological analysis.

### 4.4. Electro- and Sham Acupuncture Treatment Procedures

Ear acupuncture needles (32-gauge) were inserted into specific acupoints, and a stimulator (Ito Trio-300, Ito, Japan) was used to deliver electrical stimulation at an intensity of 2 mA at 2 Hz and a 150-microsecond pulse width. For EA treatment, Hegu (LI4) and Quchi (LI11) acupoints were selected. These acupoints were used for xerostomia treatment in previous studies [54,55]. The murine LI4 was located on the first dorsal interossei, being radial to the midpoint of the second metacarpal bone in the forelimb. Similarly, murine LI11 was located at the depression medial to the extensor carpi radialis brevis (at the lateral end of the cubital crease). The sham acupoint in sham acupuncture was determined at the middle point of the lateral deltoid muscle. 5−FU + EA and 5−FU + Sham mice were given EA or sham acupuncture, respectively, in a total of 7 sessions, for 20 min/session, on days −2, −1, 0, 1, 3, 5, and 6. The other groups only received anesthesia. 

### 4.5. Pilocarpine-Stimulated Salivary Flow Rate Measurement

Before collecting saliva, each mouse was weighed and anesthetized using an intraperitoneal (IP) injection of zolazepam/tiletamine (Zoletil 50^®^, Virbac; 40 mg/kg) and xylazine (4.6 mg/kg). After 10 min, pilocarpine (Sigma-Aldrich, St. Louis, MO, USA; 0.5 mg/kg) was injected subcutaneously in anesthetized mice to stimulate salivation. The saliva secreted for 20 min after the pilocarpine injection was absorbed into a previously weighed conical-shaped swab, which was reweighed after saliva collection. The difference between the two weights of the swab was that the weight of saliva was secreted over 20 min, while the salivary flow rate (SFR) was expressed as mg/min. The protocol was carried out as previously described [56]. The measurement of stimulated SFR was performed at the beginning of the study, to determine the baseline, and on day 7. For salivary composition analysis, the samples were centrifuged for 2 min at 7500× *g* and a temperature of 4 °C. Aliquots containing 30–60 μL of supernatants were collected and stored at −80 °C to enable further quantification of the lysozyme activity and IgA secretion rate. The experiments were completed within 14 days.

### 4.6. Quantification of Salivary Lysozyme Activity and IgA Secretion Rates via ELISA

Lysozyme activity from stimulated saliva samples was determined using an ELISA Kit (EnzChek^®^ Lysozyme Assay Kit; Molecular Probes, Eugene, OR, USA, E-22013) according to the manufacturer’s instructions. A saliva sample with a volume of 25–50 µL was used for each reaction in a fluorescence microplate. In the beginning, 50 µL of the 50 µg/mL was used. The working suspension of DQ lysozyme substrate was added to each microplate well that contained either the experimental sample or the standard curve sample. Using a fluorescence microplate reader with a fluorescein filter (Thermo Scientific Varioskan LUX, Life Technologies Holdings Pte. Ltd., Singapore), the fluorescence intensity of each reaction was measured every 5 min to record the kinetics of the reaction at 37 °C for 60 min. The absorption and fluorescence emission maxima were set at ~485 nm and ~530 nm, respectively. The values derived from the reactions after 5 min were used to determine the lysozyme activity of the experimental samples based on the standard curve. The results were shown as U/mL. 

The salivary IgA secretion rate was measured using an ELISA Kit (Invitrogen™ IgA Mouse Uncoated ELISA Kit, Cat # 88-50450-22) according to the manufacturer’s instructions. In brief, plates were coated overnight at 4 °C with pre-titrated and HRP-conjugated anti-mouse IgA polyclonal antibody 1:250 in Coating Buffer (1×PBS). Plates were washed twice with Wash Buffer and subsequently blocked at room temperature for 2 h. Next, 2-fold serial dilutions of the standards were performed to create the standard curve. Prediluted saliva samples (1:100) were added into wells in duplicate, and the plate was incubated at room temperature for 2 h on a shaker. The detection antibody was added to each well after 4 washes, and the plate was incubated at room temperature for 1 h on a shaker. After 4 washes, substrate solution (Tetramethylbenzidine–TMB) was added to each well. The reaction was stopped after 15 min using 1-molarity H_3_PO_4_ solution. We read the plate using a microplate reader (Thermo Scientific Varioskan LUX, Life Technologies Holdings Pte. Ltd., Singapore) at 450 nm and analyzed data to determine the IgA concentration of the saliva samples based on the standard curve. The IgA secretion rate was determined by multiplying the IgA concentration by the volume of stimulated saliva secreted over 20 min. The results are expressed as ng/20 min.

### 4.7. Hematoxylin and Eosin Staining and Histological Analysis

On the day of sacrifice, tissue samples of SMG were collected, fixed in 10% neutral buffered formalin, dehydrated, and embedded in paraffin. Hematoxylin and eosin (H&E) staining was performed on 4-micrometer thick sections of paraffin-embedded samples. The tissue sections were deparaffinized in xylene, subsequently rehydrated using graded ethanol (100%~75%), and briefly washed in tap water. The sections were stained in Gill II hematoxylin solution (Leica, #3801522) for 1 min and washed under running tap water for 3 min, and they were then counterstained using Eosin Y solution (Leica, #3801602) for 5 min. The sections were subsequently dehydrated using graded ethanol (95~100%), cleared in xylene, and mounted via glass coverslips using a xylene-based mounting medium. After scanning using a slide scanner (SLIDEVEW™ VS200, Olympus Corporation, Tokyo, Japan) the tissue sections were processed via its viewer software (OlyVIA 3.2, Olympus Soft Solutions GmbH, Münster, Germany). Next, 10 areas per tissue section at 200-fold magnification were randomly selected to count the number of secretory acini in the SMG. The results were expressed as the mean of the acini number/area. In addition, 20 acinar cells per tissue section were randomly selected to measure the acinar cell and nuclear diameters at 400-fold magnification. The results are expressed as µm.

### 4.8. Immunohistochemistry (IHC)

Paraffin-embedded tissue sections were deparaffinized in xylene and immersed in a descending series of alcohol solutions for rehydration. After washing in PBS buffer, we covered the sections in hydrogen peroxide blocks for 10 min. Heat-mediated antigen retrieval was performed using Tris-EDTA (pH 9.0) for 30 min. Subsequently, Immunoblock was applied, followed by incubation using the rabbit polyclonal anti-mouse AQP5 (1:100; ab78486, Abcam, Cambridge, MA, USA) at 37 °C for 1 h. After washing, primary antibody staining was detected via sequential incubation with Primary Antibody Amplifier Quanto and HRP Polymer Quanto (TL-125-QPB and TL-125-QPH, Epredia™ TL-125-QHD, Fisher Scientific; Thermo Fisher Scientific. Inc., Waltham, MA, USA) for distinct 10-min periods. DAB chromogen and substrate buffer (TA-004-QHCX and TA-125-QHSX, Epredia™ TL-125-QH) were mixed and immediately applied to tissue sections for visualization. Sections were then counterstained using hematoxylin solution. The tissue sections were mounted via a xylene-based mounting medium, and images were captured using a slide scanner (SLIDEVEW™ VS200, Olympus Corporation).

### 4.9. Evaluation of Pro-Inflammatory Cytokines TNF-α, IL-1β, and AQP5 mRNA Expression via Quantitative PCR

Fresh tissue collection: The mice were anesthetized using urethane, before being sacrificed by having their heads cut off. The murine SGs were excised, collected, and snap-frozen in liquid nitrogen, before being stored in a −80 °C refrigerator to await for RNA extraction.

Total RNA extraction from SMG tissue using the PureLink^®^ RNA Mini Kit (Ambion), was performed according to the manufacturer’s instructions. The RNA concentration was determined using a NanoPhotometer^®^ NP80 spectrophotometer (Implen, Munich, Germany). The SuperScript™ IV First-Strand Synthesis System (Invitrogen, Carlsbad, CA, USA) was used to perform reverse-transcription of RNA into complementary DNA (cDNA) according to the manufacturer’s instructions. The qPCR analysis was performed in a StepOnePlus™ Real-Time PCR System (Applied Biosystems, Waltham, MS, USA) using the 2X Universal SYBR Green Fast qPCR Mix (Abclonal, BioAb, Inc. Woburn, MA, USA, RK21203) according to the manufacturer’s protocols. The thermocycling parameters of qPCR were set at 95 °C for 3 min (Holding Stage), followed by 40 cycles at 95 °C for 5 s and 60 °C for 30 s (Cycling Stage). At the end of the amplification cycle, a melting curve was detected at temperatures between 60 and 95 °C. cDNA was amplified via PCR using forward (F) and reverse (R) primers (MDBio Inc., Taipei, Taiwan). The mouse housekeeping gene *glyceraldehyde-3-phosphate dehydrogenase (GAPDH)* was amplified in tandem with the control gene. Each sample was tested in triplicate or duplicate via qPCR. We employed the 2^−ΔΔCt^ method (Livak and Schmittgen 2001) to compare the relative quantity of gene expression to the control group. Melt curve analysis was used to confirm the specificity of the amplification. The gene’s primer forward and reverse sequences were used as listed below:

TNF-α: forward 5′-CCCTCACACTCAGATCATCTTCT-3′ and reverse 5′-GCTACGACGTGGGCTACAG-3′;

IL-1β: forward 5′-TGGACCTTCCAGGATGAGGACA-3′ and reverse 5′-GTTCATCTCGGAGCCTGTAGTG-3′;

AQP5: forward 5′-GAGGACTGGGAAGATCATAGAGAGG-3′ and reverse 5′-CAAACTCTTCGTCTTCCTTTTCTCC-3′;

GAPDH: forward 5′-TGTGTCCGTCGTGGATCTGA-3′ and reverse 5′-TTGCTGTTGAAGTCGCAGGAG-3′.

### 4.10. Statistical Analysis

Data were presented as the mean ± SD. One-way ANOVA with Tukey’s multiple comparisons test was used to analyze the experimental data. The differences were considered to be statistically significant when *p* < 0.05. All statistical analyses were conducted using GraphPrism 9 (GraphPad Software Inc., La Jolla, CA, USA).

## 5. Conclusions

We successfully created a mouse model for chemotherapy-induced salivary gland hypofunction via tail vein injection of 5−FU, in which SFR, SGW, SIgA, and lysozyme levels significantly decreased. Histological observation and RT-qPCR of SG tissues revealed significant decreases in acini numbers and AQP5 expression, while acinar cell size and TNF-α and IL-1β expression were increased. These manifestations were attenuated with EA, but not with sham acupuncture treatment, whereas pilocarpine treatment could only elevate SFR and AQP5 expression. These findings indicate that EA can prevent 5−FU-related damage to the salivary gland, providing significant insight and evidence to support EA’s use as an alternative treatment for chemotherapy-induced salivary gland hypofunction and xerostomia.

## Data Availability

The data used to support the findings of this study are available from the corresponding author upon request.
